# Computational Enzyme Engineering Pipelines for Optimized Production of Renewable Chemicals

**DOI:** 10.3389/fbioe.2021.673005

**Published:** 2021-06-15

**Authors:** Marc Scherer, Sarel J. Fleishman, Patrik R. Jones, Thomas Dandekar, Elena Bencurova

**Affiliations:** ^1^Department of Bioinformatics, Julius-Maximilians University of Würzburg, Würzburg, Germany; ^2^Department of Biomolecular Sciences, Weizmann Institute of Science, Rehovot, Israel; ^3^Department of Life Sciences, Imperial College London, London, United Kingdom

**Keywords:** computational, enzyme, engineering, design, biomanufacturing, biofuel, microbes, metabolism

## Abstract

To enable a sustainable supply of chemicals, novel biotechnological solutions are required that replace the reliance on fossil resources. One potential solution is to utilize tailored biosynthetic modules for the metabolic conversion of CO_2_ or organic waste to chemicals and fuel by microorganisms. Currently, it is challenging to commercialize biotechnological processes for renewable chemical biomanufacturing because of a lack of highly active and specific biocatalysts. As experimental methods to engineer biocatalysts are time- and cost-intensive, it is important to establish efficient and reliable computational tools that can speed up the identification or optimization of selective, highly active, and stable enzyme variants for utilization in the biotechnological industry. Here, we review and suggest combinations of effective state-of-the-art software and online tools available for computational enzyme engineering pipelines to optimize metabolic pathways for the biosynthesis of renewable chemicals. Using examples relevant for biotechnology, we explain the underlying principles of enzyme engineering and design and illuminate future directions for automated optimization of biocatalysts for the assembly of synthetic metabolic pathways.

## Introduction

At the start of the third decade of the twenty-first century, humankind faces a multitude of challenges regarding climate change (Arnell et al., 2019), air pollution (Wang et al., 2019), and a shrinking number of intact ecosystems (Nolan et al., 2018) due to human activity. The demand for sustainable solutions addressing the basis of chemical production, transport, and agriculture to enable a net zero-carbon society is higher than ever before (Hoegh-Guldberg et al., 2019). Hence, the development of technologies substituting fossil resources is an important goal of current scientific research (Xu et al., 2018). Utilizing the synthetic power of microorganisms for the sustainable production of bulk chemicals and fuels to replace chemicals currently generated from fossil fuels and tropical plant agriculture is an important contributor toward the goal of achieving a net zero-carbon society (Rodionova et al., 2017). In this regard, biosynthesis of hydrocarbons in microorganisms can be a sustainable technological alternative to produce fuels for aviation (Schirmer et al., 2010; Kallio et al., 2014a), a model of transportation for which competitive electric solutions are still missing (Schäfer et al., 2019). The benefits of applying biocatalysts in the industrial production of commodity chemicals compared to inorganic catalysts lie mostly in their ability to facilitate enantioselective conversions at ambient conditions (temperature and pressure) (Woodley, 2020). Additionally, the usage of biocatalysts instead of metal catalysts, for example, can reduce the amount of waste of chemical production because biocatalysts can be recycled easily (Sheldon and Woodley, 2018).

Further optimization of biocatalysts can expand the solution space of an enzyme and enable the identification of novel synthetic pathways for biomanufacturing of chemicals (Erb et al., 2017). Hence, engineering enzymes for tailored substrate specificity (Amer et al., 2020; Eser et al., 2020), catalytic efficiency (Risso et al., 2020), and stability (Goldenzweig et al., 2016; Yu et al., 2017) are important for the implementation of novel biosynthetic systems.

**FIGURE 1:**
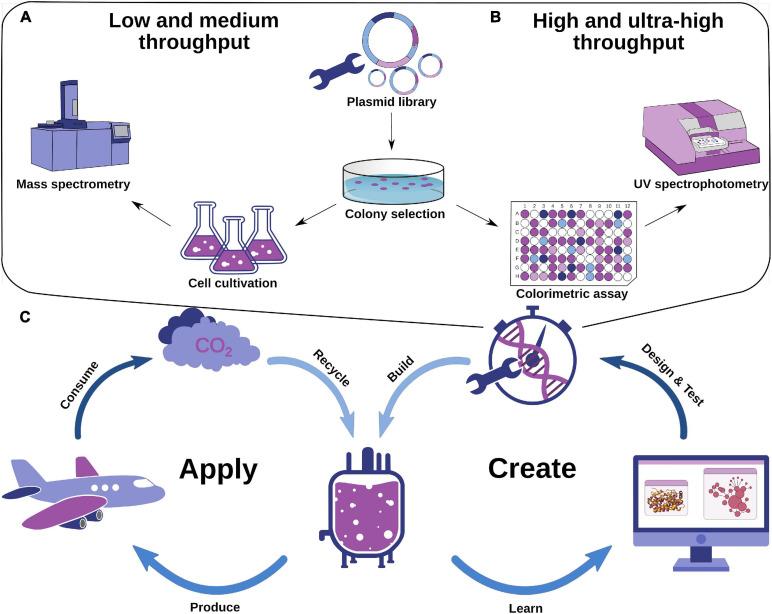
Experimental protein engineering strategies and an idealized scheme for a design–test–build–learn cycle of optimizing enzymes using computation. **(A)** Exemplified workflow for low- and medium-throughput enzyme engineering strategies. **(B)** Exemplified workflow for high- and ultrahigh-throughput enzyme engineering strategies. **(C)** Design–test–build–learn cycle for industrial chemical production including computational methodology and production–consumption–recycling cycle of chemical usage. Promising enzyme variants are identified computationally, which leads to targeted experimental testing. The metabolic systems are then applied in microorganisms for industrial-scale production. The experimental implementation provides additional information for computational optimization. Consumption of chemicals as biofuels results in the release of CO_2_, which can be recycled by microorganisms in bioreactors to close the cycle.

Efforts for enzyme engineering are exponentially growing due to the demand for natural or biologically produced chemical compounds, such as alcohols, hormones, or essential oils, either by constructing *de novo*–designed pathways or by optimizing existing ones (Marcheschi et al., 2013). In addition, current progress in genome sequencing identified a number of new enzymes or strain-specific variants that may be an alternative for the application in biotechnology; however, in a lot of cases, they are not stable or suitable for standard expression strains. Currently, enzyme engineering efforts are mostly based on rational engineering with low- and medium-throughput screening of small libraries (Figure 1A) and directed evolution-based approaches and high- and ultrahigh-throughput screening (Figure 1B; Ma et al., 2021); nevertheless, also *de novo* approaches start to get more attention and had been already used in several works (DeLoache et al., 2015; Dou et al., 2018). Interestingly, including computational tools (Romero-Rivera et al., 2017) as evolutionary conservation analysis (Ashkenazy et al., 2016), mutant structure modeling (Khersonsky et al., 2018; Leman et al., 2020), and molecular dynamics (MD) simulations (Yu and Dalby, 2018; Surpeta et al., 2020) is becoming more abundant and has the potential to accelerate the identification of highly stable and productive biocatalysts for sustainable application (Figure 1C). The development of easy-to-use software and tools available as online servers makes it possible for researchers who are not experts in computational biology to apply state-of-the-art computational protein engineering methodology. On the other hand, the data from *in silico* engineering do not necessarily correlate with experimental data (Pucci and Rooman, 2016; Carlin et al., 2017), and thus more advanced pipelines using multiple computational tools are required for accurate mutant structure modeling and energy predictions. The application of engineered proteins is versatile and covering various technological branches from pharmaceutics to bioelectronic devices (Kalyoncu et al., 2017) and biosensors (Xiong et al., 2017; Kunjapur and Prather, 2019).

Our research in synthetic biology and metabolic engineering is directed toward developing methods for bioproduction of renewable chemicals with special emphasis on biofuel-producing pathways. We and others have found that conventional strategies such as directed evolution are not applicable to all enzymatic reactions for lack of high-throughput assays that are required for the effective use of laboratory-evolution strategies. This has turned our attention to computational enzyme engineering methodology that can guide the experimental efforts. It is important to note that the modules described here can be applied with necessary adjustments to all kinds of protein engineering tasks and are therefore not limited to the field of metabolic enzyme engineering. Still, the application of computational methodology will be discussed on the example of metabolic enzymes involved in biofuel production to highlight strengths and limitations of such approaches on a particular field of biotechnological research.

**FIGURE 2:**
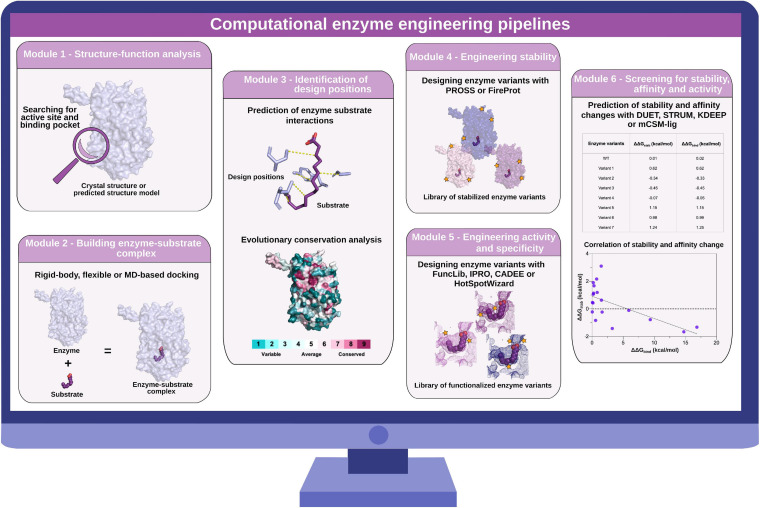
Computational enzyme engineering pipelines. Module 1: structure–function analysis to identify active site and substrate-binding pocket. Module 2: building enzyme–substrate complexes with molecular docking approaches. Module 3: identification of design positions for the subsequent sequence design. Module 4: engineering stability of enzymes with PROSS and FireProt. Module 5: engineering activity and specificity of enzymes with FuncLib, IPRO, CADEE, and HotSpotWizard. Module 6: screening for stability, affinity, and activity changes with DUET, STRUM, KDEEP, and mCSM-lig.

In this article, we review computational tools that can be used to create a platform for fast and customizable modeling and evaluation of promising enzyme variants *in silico* (Figure 2). We focus on methods that have been experimentally validated and shown to outperform conventional *in vitro* selection methods. We conclude that computational enzyme engineering can accelerate the development of synthetic metabolic pathways for industrial use.

## Metabolic Engineering of Fatty Acid Biosynthesis and Enzyme Engineering for Enhanced Production of Biofuels

**FIGURE 3:**
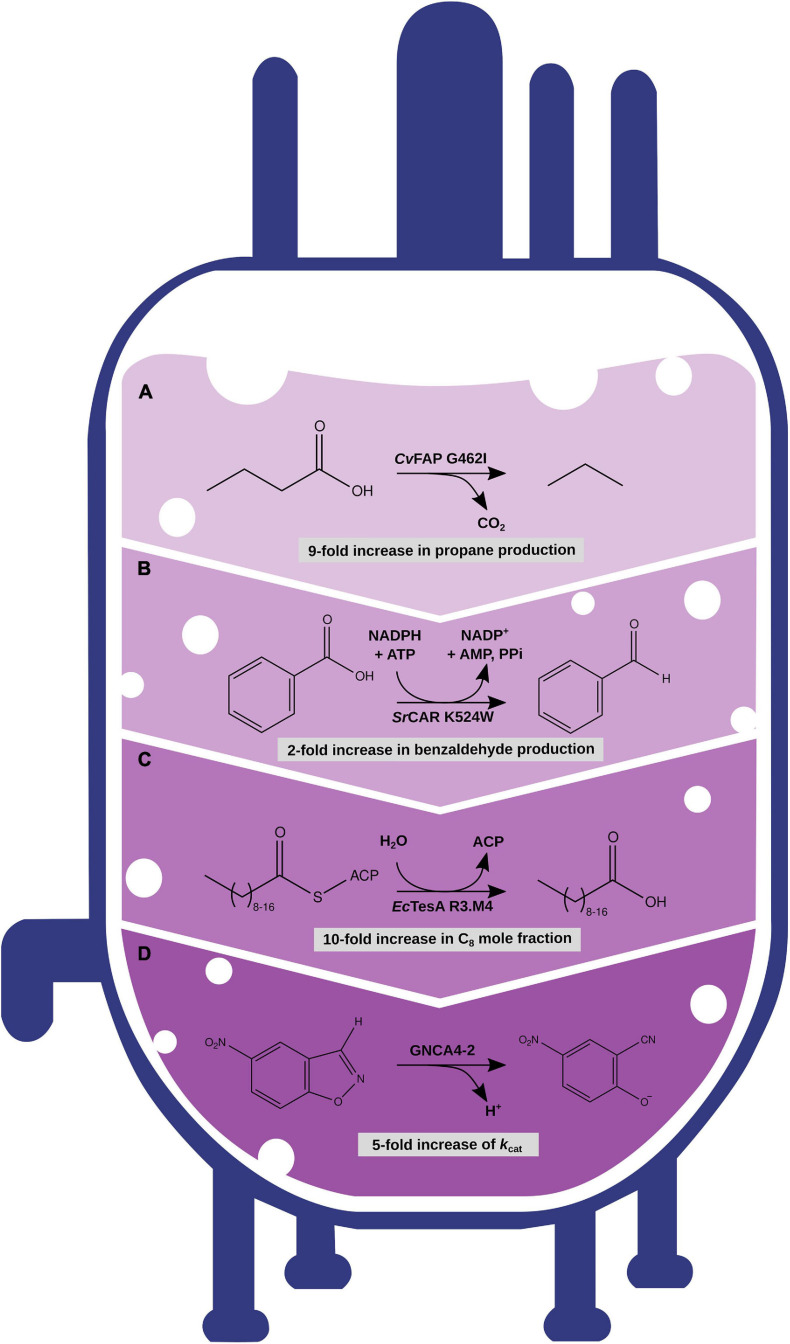
Optimization of enzymes with different engineering strategies with increasing amount of computational modeling and predictions. **(A)** Rational engineering of CvFAP enzyme for the increased production of propane. **(B)** Rational engineering based on MD simulation data of SrCAR increased production of benzaldehyde. **(C)** Semirational sequence design with IPRO of EcTesA for altered substrate specificity. **(D)** Semirational sequence design with FuncLib and subsequent screening with the EVB approach to increase the catalytic efficiency of a Kemp eliminase GNCA4.

Fatty acyl compounds are an important target for engineering microbial metabolism and chosen as example. By adding heterologous enzymatic modules, fatty acid metabolism can be redirected toward alkane/alkene biosynthesis (Liu and Li, 2020). Several metabolic pathways for the synthesis of alkanes of varying chain length have been reported (Schirmer et al., 2010; Bernard et al., 2012; Kallio et al., 2014b; Sorigué et al., 2017; Yunus et al., 2018; Amer et al., 2020). However, the production of the structurally similar class of alkenes, especially medium- and short-chain length alkenes, remains a challenge. Although first attempts at biosynthesis of medium- and short-chain alkenes have been made (Dennig et al., 2015; Zhang et al., 2019; Bauer et al., 2020), the substrate conversion efficiencies remain low. Further optimization of metabolic pathways will be required to facilitate future commercialization. The improvement of key enzymatic properties, such as stability and modified substrate specificity and activity, may be necessary, but that is traditionally a cost-intensive and time-consuming task. For example, in a study by Bao et al., single residues in the binding pocket of the *Synechococcus elongatus* cyanobacterial aldehyde-deformylating oxygenase (cADO) were targeted for site-directed mutagenesis experiments. Substitution of small residues by bulkier hydrophobic ones blocked parts of the binding pocket, which led to a shift in substrate specificity. Depending on the position of the substituted residue, specificity of the engineered cADO variants ranged from C_4_ to C_12_ substrates (Bao et al., 2016). With similar structure–function–based approaches, residues near the active site of *Chlorella variabilis* NC64A fatty acid photodecarboxylase (*Cv*FAP) (Figure 3A) and *Jeotgalicoccus* sp. ATCC 8456 OleT_JE_ were targeted recently to engineer substrate specificity of the enzymes for the short-chain-length substrate butyric acid, enabling increased production titers of propane (Amer et al., 2020) and propene (Bauer et al., 2020), respectively. All these examples have in common that a small library of rationally designed single-point or double mutants was synthesized and tested for elevated production levels and altered substrate specificities (Figure 1A). Despite these successes, however, in many cases multiple mutations are required to generate an enzyme variant with robust production titers (Grisewood et al., 2017; Khersonsky et al., 2018; Trudeau et al., 2018), and effects of enzyme destabilization upon mutations may interfere with a beneficial effect on substrate binding or catalysis (Trudeau and Tawfik, 2019). Therefore, adding computational prediction and engineering tools to the overall pipeline is likely to increase chances to identify an enzyme variant with enhanced properties. Until now, the most common method to engineer enzymes is by repetitive rounds of directed evolution-based sequence randomization and high-throughput screening (Figure 1B; Farinas et al., 2001). Such approaches are time-consuming and heavily rely on the availability of a suitable screening methodology, which has not been developed for terminal-alkene production yet (Sulzbach and Kunjapur, 2020). This emphasizes the importance of exploiting computational engineering solutions, at least in the special case of alkenes.

## Computational Pipelines to Engineer Enzymes

Advances in the fields of structural bioinformatics, computational modeling, and the availability of huge amounts of DNA sequence data have led to the development of a variety of computational tools that can speed up enzyme engineering for biotechnological application. Computational enzyme engineering and design methodology has been reviewed recently for altering properties such as stability, substrate specificity, or activity of biocatalysts (Ebert and Pelletier, 2017; Chowdhury and Maranas, 2020; Sequeiros-Borja et al., 2020). Here, we describe which enzymatic features are important for enzyme engineering and design and how recently published computational tools (Table 1) can facilitate the required steps from preparing the input structure complexes to screening designed variants for best performance. Combining these engineering steps, we propose a pipeline for computational enzyme engineering and design (Figure 2) that includes workflows for engineering stability, activity, and specificity of enzymes. Depending on the research objective, different engineering efforts can be combined to first design a stable enzyme variant that can be functionalized afterward. Advantages and disadvantages of the most promising stand-alone software and web applications are summarized in Table 2. Initially, a structure–function analysis (Module 1) is performed. Then, Module 2 describes how to build enzyme–substrate complexes. Subsequent analysis of enzyme–substrate interaction and evolutionary conservation analysis leads to the identification of design positions (Module 3). Next, the sequence space of the enzyme–substrate complex can be designed for stability engineering (Module 4) and/or activity and specificity engineering (Module 5). The pipelines end with computational stability, affinity, and activity screening (Module 6) to identify the best variants for experimental testing. Additionally, we provide information on how computational modules have paved the way and will speed up the discovery of enzyme variants for increased chemical production in the future.

**TABLE 1:** State-of-the-art software and online tools for computational protein engineering pipelines.

**Module no.**	**Method**	**Tool/software**	**References**
Module 1	Protein structure prediction	trRosetta, I-TASSER, MODELLER	Roy et al., 2010; Webb and Sali, 2016; Yang et al., 2020
	MD-based binding pocket search	TRAPP, CaverWeb1.0	Stank et al., 2017; Stourac et al., 2019
	Molecular dynamics	GROMACS, AMBER	Salomon-Ferrer et al., 2013; Abraham et al., 2015
	Protein–protein docking	GalaxyHomomer, ZING	Baek et al., 2017, 2019; Vangaveti et al., 2020
Module 2	Molecular docking	GALigandDock	Park et al., 2021
Module3	Protein–substrate interaction analysis	Arpeggio, GSP4PDB	Jubb et al., 2017; Angles et al., 2020
	Evolutionary conservation analysis	ConSurf, SMI-BLAST	Ashkenazy et al., 2016; Jin et al., 2020
	Dynamic cross-correlation analysis	GROMACS + R	Yan et al., 2000; Abraham et al., 2015; Yu and Dalby, 2020
Module 4	Protein stabilization	PROSS, FireProt	Goldenzweig et al., 2016; Musil et al., 2017
	Protein solubility prediction	Protein-sol	Hebditch and Warwicker, 2019
Module 5	Protein functionalization	FuncLib, IPRO, CADEE, HotSpotWizard3.0	Pantazes et al., 2015; Amrein et al., 2017; Khersonsky et al., 2018; Sumbalova et al., 2018
Module 6	Protein stability prediction	DUET, STRUM, DynaMut	Pires et al., 2014a; Quan et al., 2016; Rodrigues et al., 2018
	Protein–substrate binding affinity prediction	KDEEP, mCSM-lig, Rosetta flexddG	Pires et al., 2016; Barlow et al., 2018; Jiménez et al., 2018

**TABLE 2:** Advantages and disadvantages of selected state-of-the-art software and online tools for computational protein engineering.

**Module no.**	**Software**	**Function in pipeline**	**Advantages**	**Disadvantages**
Module 1	trRosetta	Obtain enzyme structure model	High accuracy of most enzyme structure models	Enzymes lacking structural and sequence similarity are often predicted with low quality scores
	GROMACS	Understand catalytic mechanism and conformational flexibility	Time-resolved sampling of overall enzyme conformations and functional residues	Computation-intensive, requirement of substrate and cofactor parameters for force field
Module 2	GALigandDock	Obtain enzyme–substrate complex	Precise sub-angstrom enzyme substrate docking	Location of binding pocket needs to be known
Module 3	ConSurf	Analyze evolutionary conservation of single residues of enzyme	Fast and easy assessment of importance of residues	Requirement of high number of homologous sequences
Module 4	PROSS	Stabilize enzyme–substrate complex	Automated identification and modeling of multipoint mutants with increased expression and stability	Requirement of enzyme structure and a few dozen homologous sequences
Module 5	FuncLib	Engineer functionality of enzyme	Automated identification and modeling of multipoint mutants for functionality screening	Requirement of enzyme structure and of a few dozen homologous sequences
Module 6	DUET	Predict stability changes of enzyme upon mutation	Fast and easy assessment of changes in stability due to mutation	Only stability changes due to single-point mutations can be predicted
	Rosetta flexddG	Predict enzyme substrate affinity changes upon mutation	Accurate prediction of changes in binding affinity due to mutations (not only single-point mutations)	Knowledge of Rosetta computing required

## Module 1: Structure–Function Analysis of Enzymes

Before starting to engineer an enzyme, a deep understanding of the structural and dynamical underpinnings of enzymatic function has to be acquired. The availability of a crystal structure is still a prerequisite for successful rational engineering that can be exemplified by the discovery of mutations in the binding pocket changing the substrate specificity of the *Cv*FAP (Figure 3A; Amer et al., 2020), the thioesterase TesA (Deng et al., 2020), and cADO (Bao et al., 2016). If the crystal structure has not been determined yet, computer-based protein structure prediction could generate models for structure–function analysis (Roy et al., 2010; Webb and Sali, 2016; Yang et al., 2020), but it is important to note that subsequent engineering heavily depends on the quality of the enzyme model (Kuhlman and Bradley, 2019). Recent advances in deep learning–based structure prediction are likely to change this situation completely, alleviating step-by-step the need for a crystal structure (Senior et al., 2020; Singh, 2020; Torrisi et al., 2020). The field of *ab initio* structure prediction has recently advanced tremendously through deep learning methods and that its accuracy even surpasses that of high-quality homology modeling and reaches the point of atomic accuracy. With this, it is quite likely that such models would be good starting points for protein design calculations, circumventing laborious structure determination by experimental methods.

In cases where the exact location of the binding pocket and active site of an enzyme is not known, MD simulation-based approaches can be applied to analyze substrate-binding trajectories and identify hidden binding pockets (Petřek et al., 2006; Stank et al., 2017; Stourac et al., 2019). The Caver 1.0 webserver, for example, was used to identify residues forming the access tunnel and substrate-binding cavity of the *Serratia marcescens* prodigiosin ligase PigC. Subsequent targeting of these residues in mutagenesis experiments revealed a double mutant with a shift in substrate preference by enhancing the catalytic efficiency (*k*_cat_) 3.4-fold for the pharmaceutically interesting short-chain prodiginines compared to the wild-type PigC (Brands et al., 2021).

Furthermore, understanding the catalytic mechanism of an enzyme can be helpful (Chen and Arnold, 2020). Simulating the MD of *Segniliparus rugosus* carboxylic acid reductase (*Sr*CAR) (Qu et al., 2019a), which catalyzes the reduction of carboxylic acids to the corresponding aldehydes (Winkler, 2018; Figure 3B), sheds light on the structural underpinnings of catalytic mechanism and conformational flexibility. The information acquired by molecular dynamic simulation enabled the discovery of an *Sr*CAR single-point mutant that showed an increase in enzymatic activity for benzoic acid by twofold (Qu et al., 2019b). This example highlights how studying the dynamic nature of catalysis in enzyme engineering can be used for optimization strategies.

Many enzymes form complexes by the assembly of protein subunits or monomers (Levy and Teichmann, 2013), as recently discovered for *Cv*FAP (Lakavath et al., 2020), or interactions with additional molecules as cofactors to facilitate chemical conversions (Kara et al., 2014). Therefore, it is important to elucidate which residues are essential for such molecular interactions to restrain residue positions during the engineering process. Protein–protein interfaces and multimerization can be predicted by protein–protein docking (Baek et al., 2017, 2019; Vangaveti et al., 2020).

## Module 2: Building the Enzyme–Substrate Complex

Modeling enzyme variants in the presence of the cognate substrate has shown to be helpful for both activity and specificity engineering (Jha et al., 2015; Grisewood et al., 2017; Risso et al., 2020). Hence, obtaining an enzyme–substrate complex with accurate substrate-binding poses or transition state complex is the first step in the proposed computational pipeline. Molecular docking is a widely used method in drug discovery for identification of substrate-binding poses by searching the conformational space for the best fit to the binding pocket of the protein (Pagadala et al., 2017) and can be applied similarly in enzyme engineering (Ebert and Pelletier, 2017). First molecular docking algorithms mainly focused on docking small molecules into a single static structure of a protein (rigid-body docking), hence immensely reducing the complexity of computation under the penalty of precision (Kuntz et al., 1982; Ebert and Pelletier, 2017). Over the years, molecular docking has advanced to take the dynamic nature of substrate binding and the conformational flexibility of enzymes into account (Pinzi and Rastelli, 2019). Such flexible docking (Carlson, 2002; Davis and Baker, 2009; Forli et al., 2016) or unbiased (Shan et al., 2011) and biased (Ebert et al., 2017; Kokh et al., 2020) MD-based docking approaches benefit from docking the substrate into an ensemble of structures rather than one single structure (static), therefore describing substrate-binding more accurately. Current methods in rigid-body, flexible, and MD-based substrate docking were compared elsewhere (Kotev et al., 2016). Still, engineering or design of an enzyme–substrate complex requires almost atomic-level accuracy, which may not be reached with the molecular docking methods mentioned previously. Recently, a novel approach called GALigandDock was reported that enabled small molecule docking with sub-angstrom accuracy in the Rosetta framework by force field optimization (Park et al., 2021). GALigandDock outperformed the top current docking approaches in a cross-docking benchmark set providing new directions for automated enzyme–substrate complex modeling.

## Module 3: Identification of Design Positions

After the enzyme–substrate complex has been prepared, the next step is to identify residues (design positions or hotspots) that are allowed to mutate during the sequence design steps (see Modules 4 and 5). Computational tools that predict prominent interactions between enzyme and substrate can be used to analyze the basic molecular interaction network in the binding pocket (Jubb et al., 2017; Angles et al., 2020). A prediction of the importance of single residues or structural motifs can be achieved based on an evolutionary conservation analysis (Ashkenazy et al., 2016; Gil and Fiser, 2019; Jin et al., 2020), which can be helpful to assess suitability of residues as design positions. The ConSurf webserver for example was used to guide the engineering of PigC (see Module 1) to exclude those residues from mutagenesis experiments that showed high conservation scores as they are likely to be essential for enzymatic function (Brands et al., 2021). Depending on the engineering task, altering polarity and/or size of binding pocket residues has to be considered to increase activity or change specificity of the enzyme. It is important to keep in mind that changes in activity and substrate specificity are not always connected to mutations of the first shell. It has been shown that second shell mutations or even remote mutations can modulate functionality of enzymes (Trudeau et al., 2018; Wilding et al., 2019; Osuna, 2020). Interaction networks that may span the whole size of the enzyme facilitate conformational flexibility. With the aid of MD simulations and dynamic cross-correlation network analysis, such interaction networks can be identified and targeted for engineering (see also Module 4; Yu and Dalby, 2018, 2020). Additionally, biased MD simulations can be used to identify mutational hotspots by simulating substrate binding as shown for the cytochrome P450 enzyme CYP102A1 (BM3) binding palmitic acid (Ebert et al., 2017). In this study, simulating the steered movement of the substrate toward BM3 revealed all residues involved in substrate binding even a formally unknown Q73 of the second shell of the binding pocket. Its importance was proven by site-directed mutagenesis revealing a single-point mutant that showed a fivefold increase of the Michaelis constant (K_M_) compared to the wild-type BM3.

## Module 4: Engineering Stability of Enzymes

For the engineering of protein stability, several strategies and computational tools have been reviewed (Liu et al., 2019). In general, engineering an enzyme to increase expression and thermostability represents a versatile starting point to diversify enzymatic function. The introduction of mutations into the wild-type structure is likely to interfere with configurational stability and hence functionality if a certain stability threshold is exceeded (Trudeau and Tawfik, 2019). From a biotechnological viewpoint, engineering the stability of a biocatalyst can improve its robustness against elevated temperature or organic solvents, which makes it possible to accelerate chemical conversion rates at industrial scale. Additionally, these enzymes may withstand the introduction of mutations that enhance enzymatic activity and specificity for a given substrate of interest.

To address this challenge, a combination of phylogenetic analysis and the outstanding capacity of the Rosetta software (Leaver-Fay et al., 2011) to screen and design conformational space of an enzyme scaffold enabled the identification of mutations that increased thermostability and expression levels. One application that was developed also as a webserver is called PROSS (Goldenzweig et al., 2016). PROSS uses sequence alignment to exclude mutations without occurrence in homologs sequences followed by modeling of mutant structures and Rosetta energy calculations of the native state to identify fewer than 10 candidates for experimental testing. Protein variants designed by PROSS showed dramatically increased heterologous protein expression yields underlining the ability of PROSS to optimize enzymes for heterologous expression in industrial microbial hosts as *Escherichia coli* (Goldenzweig et al., 2016). In a recently published study, PROSS was used to design three variants of the HIV-1 envelope glycoprotein gp140 with 17–45 mutations (Malladi et al., 2020). The gp140 variants maintained similar antigenicity profiles as the wild type and showed fourfold and twofold increases in protein yield, respectively. Additionally, PROSS stabilization was shown to be affective in designing enzyme variants of acetolactate synthase (AlsS) from *Bacillus subtilis* for increased solubility in 8% isobutanol while maintaining ∼80% of activity during a 5-day experiment (Sherkhanov et al., 2020). In this case, three mutant variants containing 20, 37, and 71 mutations, with and without three deletions (six mutant variants in total), respectively, were tested to identify one with increased solubility while maintaining activity in 8% isobutanol. These two examples highlight the applicability of PROSS-based protein design in biomedicine and biotechnology.

Alternatively, the FireProt webserver uses a similar approach that combines screening of stability changes (energy-based mutations) and back-to-consensus analysis (evolution-based mutations) that are summarized in a user-specific mutant library (Musil et al., 2017). FireProt was used, for example, to engineer a ketoreductase ChKRED12 for enhanced thermostability (Liu Y. et al., 2021). In this case, FireProt identified 12 (energy-based) and 17 (evolution-based) single-point mutations of ChKRED12, respectively. While the multipoint variants containing all identified mutations showed no activity, screening of 12 single-point mutants predicted with the evolution-based approach revealed four mutants with higher residual activity after 1.5 h of heat treatment at 50°C.

Both approaches, PROSS and FireProt, rely on Rosetta modeling and energy calculations but also show some differences as PROSS includes an option for specifying regions that will be excluded from the calculations as active sites or protein–protein interfaces. Additionally, PROSS designs a small library of multipoint mutants, whereas FireProt identifies a larger library of single-point mutations for experimental testing (Goldenzweig et al., 2016; Musil et al., 2017). To visualize changes in enzyme surface polarity due to the introduction of stabilizing mutations by PROSS and FireProt, the Protein-Sol web tool can be used (Hebditch and Warwicker, 2019).

An alternative strategy for stability engineering that requires a higher degree of computational skills is based on MD simulations and subsequent dynamic cross-correlation network analysis (Yu and Dalby, 2020). This approach was used to generate multipoint mutants of the *E. coli* transketolase (TK) based on four single mutations and a double-mutant variant (Yu and Dalby, 2018). The final quadruple mutant showed 10.2-fold increase in residual activity after 1-h incubation at 60°C.

## Module 5: Engineering Activity and Specificity of Enzymes

While the search for stabilizing mutations takes the whole amino acid sequence of an enzyme into account (with exceptions), rational and semirational engineering of functionality focuses on residues that are directly or indirectly connected to the catalytic center or binding pocket of an enzyme. Assuming a simplistic correlation between binding pocket shape and the ligand structure based on polarity and non-covalent bonding, redesigning these properties allows the acceptance of novel substrates to bind to the enzyme and ideally being processed as the native substrate. This has been shown for substrate and activity engineering of several enzymes connected to biofuel production (see above). Of course, the reality is much more complex, and, in many cases, multiple mutations are required to substantially increase the activity toward a novel substrate (Grisewood et al., 2017; Khersonsky et al., 2018; Trudeau et al., 2018). Computational tools have been developed that target such a goal with sequence design algorithms and subsequent ranking of the energetics of enzyme variants (Pantazes et al., 2015; Amrein et al., 2017; Khersonsky et al., 2018; Lapidoth et al., 2018; Sumbalova et al., 2018). With the aid of the IPRO (iterative computational protein library redesign and optimization procedure) algorithm (Pantazes et al., 2015), for example, scientists were able to redesign the *E. coli* thioesterase TesA, which originally showed promiscuous activity to a wide range of substrates (Figure 3C). After four rounds of sequence design with IPRO, 54 TesA variants were screened experimentally, 3 and 27 of them showed an increased mole fraction of C_12_ and C_8_ fatty acids compared to the wild-type TesA, respectively (Grisewood et al., 2017).

Alternatively, the FuncLib webserver has been used recently to engineer the substrate specificity of the *Salmonella enterica* acetyl-CoA synthetase (ACS). Screening of 29 ACS designs revealed one multipoint mutant with five mutations in or adjacent to the binding pocket that showed increased specificity for the desired substrate glycolate by twofold and decreased specificity for the native substrate acetate by eightfold at the same time (Khersonsky et al., 2018; Trudeau et al., 2018). The FuncLib algorithm is based on a four-step workflow starting with a screening of the sequence space of the design positions of an enzyme input to allow only amino acid substitutions with at least a modest probability of occurrence in nature. Second, a mutant library in which all multipoint mutants of the allowed mutations is enumerated in Rosetta. The algorithm ranks the candidates after their stability and destabilizing mutations are excluded. To ensure the generation of a diverse set of multipoint mutants, the design candidates are compared based on the introduced mutations, and if similar designs occur, the less stable version(s) is (are) excluded to avoid clustering (Khersonsky et al., 2018), resulting in a condensed library (typically of a few dozen designs) for experimental screening or further pruning through additional computational steps. Forty-nine multipoint mutant variants of the phosphotriesterase (PTE) from *Pseudomonas diminuta* were tested, and 35 variants showed an increase in esterase efficiency up to 1,000-fold. As a more radical approach to engineer/design enzymes, the applicability of automated modular backbone assembly was recently reported to generate highly active enzymes with diverse substrate preferences (Lapidoth et al., 2018) and hence represent a platform for future directions of enzyme engineering and design. In both applications, FuncLib and modular assembly, no enzyme–substrate or transitions state complex was required.

An alternative web application, the HotSpot Wizard 3.0 webserver (Sumbalova et al., 2018), comprises a large number of computational tools for modeling and assessment of protein mutations for the generation of smart libraries. Again, Rosetta software is included to calculate energy changes upon mutations to select those mutations that have a stabilizing effect on the enzyme structure. Interestingly, with the third version of HotSpot Wizard, tools for enzyme structure prediction and model quality assessment were added to increase applicability for the vast amount of enzyme sequence data whose structure has not been determined yet. Success for the application of HotSpot Wizard 3.0 in enzyme engineering has been demonstrated recently on the examples of engineering the kinetic stability of a hyperthermostable β-mannanase (Liu Z. et al., 2021) and enzymatic efficiency of a lytic polysaccharide monooxygenase MtC1 (Guo et al., 2020).

Besides the computational methods described previously that can be classified as semirational engineering strategies, a tool for computer-aided directed evolution of enzymes (CADEE) has been reported (Amrein et al., 2017). The method connects automated high-throughput mutagenesis with computational screening based on the empirical valence bond (EVB) approach to identify substitutions that change the energetics of enzymatic activation barriers and therefore might increase activity for a given chemical reaction. On the downside, CADEE needs a calibrated reference state based on experimentally tested mutations to rigorously parameterize the EVB force field for high-quality prediction (Amrein et al., 2017). Interestingly, the EVB approach was successfully applied to identify variants of a *de novo* Kemp eliminase enzyme (Figure 3D) generated by FuncLib (Risso et al., 2020; see also Module 6).

Such pipelines in which fast bioinformatics and macromolecular modeling calculations are used to select designs for intensive but accurate transition-state modeling can be an important next step that increases the accuracy of enzyme-design methodology. The enzyme engineering applications described above represent a variety of approaches that can be used to engineer novel highly active and selective biocatalysts *in silico* for industrial fuel biomanufacturing.

## Module 6: Screening for Stability, Affinity, and Activity

Depending on the approach chosen in Modules 4 and 5, the generated library of enzyme variants may exceed the size that can be screened experimentally. Furthermore, designing a diverse set of multimutation variants may require expensive gene synthesis when experimental mutagenesis will be too time-consuming. Therefore, computational screening of enzyme stability, substrate-binding affinity, or activity can speed up the process of identifying the best candidates for experimental testing and simultaneously decrease the research expenses. Nevertheless, prediction of the exact biophysical properties of an enzyme in terms of changes in stability (Pucci et al., 2018; Montanucci et al., 2019) and ligand-binding affinity (Wang et al., 2021) upon mutation are still a great challenge and therefore require intensive computation.

A variety of webservers were reported for fast prediction of stability changes upon single-point mutations mainly applying machine-learning approaches (Cheng et al., 2006; Pires et al., 2014a,b; Quan et al., 2016). A review comparing the most common machine learning–based approaches for protein stability prediction upon mutations was published recently (Fang, 2020). The impact of point mutations on dynamics of a protein and stability are provided by the DynaMut webserver (Rodrigues et al., 2018). These webservers can be useful for rational engineering of enzymatic function to screen single mutations for stabilizing effects. In contrast to the webservers mentioned previously, applications such as FireProt, PROSS, and FuncLib have an internal stability ranking with the Rosetta force field included. Thus, additional screening for stability changes is not necessary.

Similarly, the precision of applications for predicting the impact of mutations on binding affinity is growing steadily. A study by Aldeghi et al. (2018) compared different protein–ligand binding affinity prediction approaches based on a challenging benchmark set and showed that the Rosetta protocol (*flex_ddG*) (Barlow et al., 2018), although originally developed for prediction of protein–protein binding affinity changes upon mutations, produced comparable results to computation-intensive free energy calculations (Wang et al., 2021). A combination of both further increased the precision of the approach. Additionally, several webserver applications such as mCSM-lig (Pires et al., 2016) or KDEEP (Jiménez et al., 2018) were designed for the fast prediction of binding affinity changes upon single-point mutations based on machine learning algorithms.

Besides predictions of binding affinity changes, calculations of activation free energy changes of the transition state with an EVB approach proved to be useful for screening multipoint mutants of a Kemp eliminase GNCA4 designed by FuncLib (Risso et al., 2020). In this study, the 20 top-ranked GNCA4 variants were tested, and 4 variants showed enhanced *k*_cat_ values of up to one magnitude compared to the GNCA4 WT (Figure 3D).

Still, accurate prediction on protein–ligand binding affinity changes upon mutations, especially if multiple mutations are involved, remains computationally demanding and requires a high amount of knowledge of the underlying methodology and biophysical parameters of ligand binding and enzymatic catalysis. Nevertheless, future improvements of computational enzyme engineering software led by a growing amount of experimental data and advanced machine learning–based predictions will further simplify and speed up the *in silico* analysis of mutant enzyme models, similarly, modern methods in drug design and drug delivery will speed up pharmacological applications.

## Conclusion and Future Directions

Computational protein engineering promises a fast and efficient identification of enzyme variants with altered properties tailored for sustainable biomanufacturing of chemicals compared to experimental engineering techniques. The need for stability, specificity, and activity optimized biocatalysts drives the improvement of software along the engineering pipelines presented here. Still, proof of concept for the utilization of generalizable engineering pipelines including enzyme structure prediction, design, screening, and characterization of enzyme variants *in silico* is missing. The Rosetta modeling suite included in webserver applications and local software packages presents the option to perform and automate most tasks required in computational enzyme engineering by only one software. Implementation of automated molecular docking and screening platforms in webserver applications such as FuncLib could further increase the performance of Rosetta-based sequence design. The continuously increasing accuracy of protein structures prediction by machine learning–based approaches propels the development of fast and precise enzyme modeling and engineering pipelines to complement experimental engineering methodology or even open up the optimization of biocatalysts for which experimental techniques are missing. Complementing computational enzyme engineering with MD simulations provides powerful means to understand how mutations are affecting substrate binding and enzymatic activity. In the future, such computational approaches will accelerate the discovery of optimized protein-based solutions in general and of biocatalyst in particular for biotechnological and biomedical application. The computational pipelines described here can help to overcome the experimental limitations in metabolic pathway optimization on a protein level to enable, for example, industrial scale production of biofuels.

## Author Contributions

MS wrote the article. SF critically revised the article. EB, PJ, and TD contributed to the design and writing of the manuscript. All authors discussed the results and contributed to the final manuscript.

## Conflict of Interest

SF is a named inventor on patent filings regarding the PROSS and FuncLib methods and several proteins designed using these tools. The remaining authors declare that the research was conducted in the absence of any commercial or financial relationships that could be construed as a potential conflict of interest.
